# Using different anthropometric indices to assess prediction ability of type 2 diabetes in elderly population: a 5 year prospective study

**DOI:** 10.1186/s12877-018-0912-2

**Published:** 2018-09-17

**Authors:** Jing Yang, Fei Wang, Jing Wang, Xu Han, Hua Hu, Caizheng Yu, Jing Yuan, Ping Yao, Xiaoping Miao, Sheng Wei, Youjie Wang, Weihong Chen, Yuan Liang, Huan Guo, Xiaomin Zhang, Dan Zheng, Yuhan Tang, Handong Yang, Meian He

**Affiliations:** 1 Department of Occupational and Environmental Health and State Key Laboratory of Environmental Health for Incubating, School of Public Health, Tongji Medical College, Huazhong University of Science and Technology, 13 Hangkong Rd, Wuhan, 430030 Hubei China; 2 Department of Epidemiology and Biostatistics, School of Public Health, Tongji Medical College, Huazhong University of Science and Technology, Wuhan, China; 3 Dongfeng Central Hospital, Dongfeng Motor Corporation and Hubei University of Medicine, Shiyan, Hubei China

**Keywords:** Anthropometric indices, Elderly Chinese, Epidemiology, Diabetes mellitus, Prediction ability

## Abstract

**Background:**

Emerging studies have investigated the association between different anthropometric indices with diabetes risk but the results were inconsistent. The aims of the study were to examine the associations of different anthropometric indices with incident diabetes risk and whether novel anthropometric indices improve diabetes prediction beyond traditional indices among elderly Chinese.

**Methods:**

Nine thousand nine hundred sixty-two elderly individuals (age ≥ 60 years old) derived from the prospective Dongfeng-Tongji cohort were included. Hazard ratio (HR) and corresponding 95% confidence interval (CI) were evaluated by Cox proportional hazard model to examine the associations between traditional anthropometric indices (body mass index [BMI], waist circumference [WC], waist-to-height ratio [WHtR]), novel anthropometric indices (visceral adiposity index [VAI], a body shape index [ABSI], body roundness index [BRI]) and diabetes risk. Receiver operating characteristic (ROC) curve and area under curve (AUC) were applied to compare the novel anthropometric indices with the traditional indices in diabetes prediction.

**Results:**

During mean 4.6 years of follow-up, 614 incident cases of type 2 diabetes (T2D) were identified. Significant positive associations were detected between BMI, WC, WHtR, VAI and BRI and incident T2D risk. For ABSI, no significant association was observed in either men or women. BMI was the strongest predictor in diabetes in men (AUC = 0.655) comparable with the other anthropometric indices (*P* < 0.05). Similar as men, BMI was the strongest predictor (AUC = 0.635) in women. Except for WC, the AUC of BMI was larger than WHtR, VAI, and BRI. In contrast, ABSI was not a good predictor in either men (AUC = 0.507) or women (AUC = 0.503).

**Conclusions:**

In elderly Chinese, BMI, WC, WHtR, VAI and BRI were positively associated with incident T2D risk. Among them, BMI was the strongest predictor in both men and women.

**Electronic supplementary material:**

The online version of this article (10.1186/s12877-018-0912-2) contains supplementary material, which is available to authorized users.

## Background

In recent decades, type 2 diabetes (T2D) has increased rapidly and become a serious public health problem worldwide [1]. In China, the prevalence of T2D has risen to be 11.6% and over 100 million adults were affected [2].

Obesity is well recognized to be an important risk factor for the development of T2D. In epidemiological studies, anthropometric indices have been used to evaluate obesity for their simplicity and usefulness. Body mass index (BMI) has been the most commonly used anthropometric measure for defining obesity recommended by World Health Organization (WHO) [3] .Subsequently, studies found that BMI, an estimate of general obesity, could not reflect abdominal fat. Therefore waist circumference (WC) and waist-to-height ratio (WHtR) were suggested as an indicator of central adiposity [4–6], although they could not discriminate visceral fat from subcutaneous fat. In recent years, novel anthropometric indices, such as visceral adiposity index (VAI), a body shape index (ABSI), and body roundness index (BRI) have been proposed to be alternative indicators of obesity. VAI, an indicator of visceral fat dysfunction, has been reported to distinguish visceral fat from subcutaneous fat [7]. Bozorgmanesh et al. found VAI has a good predictive performance on diabetes in Tehran people [8]. In 2012, Krakauer developed a new anthropometric index named ABSI, and found ABSI was significantly associated with mortality [9]. In the following years, Thomas et al. developed another new anthropometric index known as BRI [10]. A recent study demonstrated that BRI was a potential and alternative obesity measure in assessment of T2D [11]. However, till now no comprehensive consensus has been reached about which one as the best anthropometric index to evaluate the risk and the predictive ability of diabetes, especially in elderly population.

The World Health Organization estimated that there were 600 million people aged 60 years or older in 2000, and that this number will increase to 1.2 billion in 2025 [12]. It is known that aging was related to significant changes in body composition, but it is unclear which anthropometric index is the best one to evaluate obesity and predict diabetes risk [13]. In the present study, based on the ongoing prospective Dongfeng-Tongji cohort study, we aim to examine the associations of different anthropometric indices with diabetes risk and to investigate whether these novel anthropometric indices could improve predictive ability of diabetes beyond traditional indices in elderly population.

## Methods

### Study participants

The participants in the present study were derived from the Dongfeng-Tongji (DFTJ) cohort study, which has been described elsewhere [14]. In brief, DFTJ, initiated in 2008, is a dynamic cohort study including 27,009 retirees from the Dongfeng Motor Corporation. The participants completed an epidemiology questionnaire including socio-demographic, lifestyle and medical history at baseline. The first follow-up was conducted from April to October 2013. As shown in (Additional file 1: Figure S1), participants with age below 60 years old (*n* = 7932), prevalent diabetes (*n* = 4344), coronary heart disease (*n* = 2607), stroke (*n* = 437) or cancer (*n* = 555) at baseline were excluded. Additionally, individuals were also ruled out if they had missing information on BMI (*n* = 292), WC (*n* = 56), triglyceride (TG, *n* = 766) or high-density lipoprotein cholesterol (HDL-c, *n* = 58). After exclusion, a total of 5998 men and 3964 women were eligible for the present study.

### Anthropometric measurement

Height and weight were measured without shoes and heavy clothes, using standard device and methods, recorded to the nearest 0.5 cm and 0.1 kg. WC was determined at midway level between the lower rib margin and the iliac-crest at minimal respiration. BMI was calculated as weight (kg) divided by height (m) squared. WHtR was calculated as dividing WC (cm) by height (cm). VAI was defined as the following formula [7]:

$$ \mathrm{Male}: VAI=\left(\frac{WC(cm)}{39.68+\left(1.88\times BMI\right)}\right)\times \left(\frac{TG\left( mmol/L\right)}{1.03}\right)\times \left(\frac{1.31}{HDL-c\left( mmol/L\right)}\right) $$

$$ \mathrm{Female}: VAI=\left(\frac{WC(cm)}{39.58+\left(1.89\times BMI\right)}\right)\times \left(\frac{TG\left( mmol/L\right)}{0.81}\right)\times \left(\frac{1.52}{HDL-c\left( mmol/L\right)}\right) $$

ABSI was defined as: *ABSI=WC*(m)*/ (BMI*^*2/3*^ *× height*(m)^*1/2*^*)*. BRI was defined as: *BRI = 364.2–365.5× {1 – [(WC*(m)*/2π)/ (0.5 × height*(m)*)]* ^*2*^*}*^*1/2*^.

### Type 2 diabetes definition

Type 2 diabetes cases were defined as self-reported physician-diagnosed diabetes or taking diabetes medications (oral hypoglycemic agent or insulin) or fasting glucose concentration (FBG) ≥ 7.0 mmol/L according to the WHO criteria [15]. In the study, a total of 614 incident diabetes were diagnosed.

### Covariates assessment

Overnight fasting blood specimens were obtained and FBG level was measured by Abbott Aroset analyzer. TG, TC, HDL-c and high-density lipoprotein cholesterol (LDL-c) levels were measured by ARCHITECT Ci8200 automatic analyzer (ABBOTT Laboratories. Abbott Park, Illinois, U.S.A). Smokers were defined as those who smoke at least one cigarette per day for more than half a year, and smoking status was classified as never smoking, current smoking, and former smoking. Similarly, drinkers were defined as those who drink at least once per week for more than half a year, and drinking status was classified as never drinking, current drinking, and former drinking. Physical activity was defined as those who exercise at least 20 min per time regularly over the past 6 months. Education status was categorized into two levels: low level (middle or primary school or below) and high level (high school or beyond). Hypertension was defined if blood pressure ≥ 140/90 mmHg, or using antihypertensive medication or self-reported physician-diagnosed hypertension. Hyperlipidemia was defined if TC > 5.72 mmol/L or TG > 1.70 mmol/L or using lipid-lowing medication, or self-reported physician-diagnosed hyperlipidemia.

### Statistical analysis

Categorical variables were presented in number (percentage) and continuous variables in mean (SD) or median. Student’s *t*-test, Mann-Whiney U test or Chi-square test were used for comparison between groups. In the present analysis, central obesity was defined as WC ≥85 cm in men and WC ≥ 80 cm in women. The cut-off point of WHtR was 0.5 [16]. According to the China criterion, BMI < 24 kg/m^2^ was defined as normal, 24–27.9 kg/m^2^ overweight, and BMI ≥ 28 kg/m^2^ obesity. VAI, ABSI and BRI were stratified into sex-specific tertiles. Partial correlation was used to examine the linear relationship between various anthropometric indices after adjustment for age. Cox proportional hazard model was used to calculate hazard ratio (HR) and corresponding 95% confidence intervals (CIs) of incident diabetes for different anthropometric indices. Receiver operating characteristic (ROC) analysis was used to compare discrimination ability and determine optimal cut-off value. Sensitivity and specificity were calculated based on cut-off values, which were estimated using the maximized Youden index. The areas under the ROC curves (AUC) was compared by a non-parametric test [17]. Statistical analyses were performed with SPSS Statistics 23.0 (SPSS, Chicago, Illinois, USA), except for the ROC analysis, which was tested using MedCalc V.17.9 (MedCalc Software, Belgium). A two-side value of *P* < 0.05 was considered statistically significant.

## Results

### Characteristics of participants

During mean 4.6 years of follow-up, 365 incident cases of T2D in the 5998 men (14.09/1000 person-years) and 249 cases in the 3964 women (14.45/1000 person-years) were identified. Baseline characteristics of the 614 (6.2%) participants who did and 9348 (93.8%) who did not develop to T2D are shown in Table 1. The average age of participants who did and who did not develop to T2D was 66.44 and 66.81 years old respectively. As expected, participants who developed to T2D were more likely to have higher levels of WC, WHtR, BMI, VAI, BRI, diastolic blood pressure (DBP), systolic blood pressure (SBP), TG, FBG (all *P* < 0.001), TC (*P* = 0.019) and lower levels of HDL-c (*P* = 0.001). Moreover, the rate of drinking (*P* = 0.02) in participants who developed to T2D was higher than who did not. No significant difference was observed in ABSI (*P* = 0.441), smoking (*P* = 0.854), physical activity (*P* = 0.213), education level (*P* = 0.244), and family history of T2D (*P* = 0.09) between participants who did and who did not develop to T2D.

**Table 1:** Baseline characteristic of the study population

Variables	Non T2D cases (*n* = 9348)	Incident T2D cases (*n* = 614)	*P* value
Age (years)	66.81 ± 5.55	66.44 ± 5.16	0.083
Female (%)	3715(39.7)	249(40.6)	0.690
WC (cm)	83.01 ± 9.31	87.13 ± 9.62	< 0.001
WHtR	0.51 ± 0.06	0.54 ± 0.06	< 0.001
BMI (kg/m^2^)	24.22 ± 3.32	25.86 ± 3.18	< 0.001
VAI	1.55 ± 1.31	1.93 ± 1.58	< 0.001
ABSI	0.078 ± 0.006	0.078 ± 0.006	0.441
BRI	3.69 ± 1.15	4.17 ± 1.21	< 0.001
Smoking (Yes, %)			
Current smoker	2300(24.8)	147(24.1)	
Former smoker	1319(14.2)	84(13.7)	
Never smoker	5672(61.0)	380(62.2)	0.854
Alcohol consumption (Yes, %)			
Current drinker	147(24.1)	169(27.5)	
Former drinker	84(13.7)	49(8.0)	
Never drinker	380(62.2)	396(64.5)	0.020
Physical activity (Yes, %)	8470(90.0)	547(89.1)	0.213
Education (High school or beyond, %)	3036(32.8)	186(30.5)	0.244
HDL-c (mmol/L)	1.40	1.35	0.001
TG (mmol/L)	1.15	1.36	< 0.001
TC (mmol/L)	5.10	5.17	0.019
SBP (mmHg)	129.76 ± 18.28	133.54 ± 17.72	< 0.001
DBP (mmHg)	77.35 ± 10.84	79.07 ± 10.81	< 0.001
Hypertention (Yes, %)	3739(40.0)	328(53.4)	< 0.001
Hyperlipidemia (Yes, %)	4229(45.2)	353(57.5)	< 0.001
FBG (mmol/L)	5.54 ± 0.56	6.05 ± 0.63	< 0.001
T2D family history (Yes, %)	169(1.8)	17(2.8)	0.090

Continuous variables were presented as mean ± SD or median. Categorical variables were presented as a number (percentage)

*Abbreviations*: *WC* waist circumstance, *WhtR* waist-to-height ratio, *BMI* body mass index, *VAI* visceral adiposity index, *ABSI* a body shape index, *BRI* body round index, *HDL-c* high-density lipoprotein cholesterol, *LDL-c* low-density lipoprotein, *SBP* systolic blood pressure, *DBP* diastolic blood pressure, *TG* triglyceride, *TC* total cholesterol, *FBG* fasting blood glucose, *T2D* type 2 diabetes

The correlation coefficients between various anthropometric indices are shown in (Additional file 1: Table S1). All anthropometric indices showed significant correlation with each other (*P* < 0.001). The strongest correlation coefficient was found between WHtR and BRI (*r* = 0.87 and *r* = 0.83) and the weakest one between BMI and ABSI (*r* = − 0.047 and *r* = − 0.161). Similar findings were observed in both men and women.

### Association of anthropometric indices with incident T2D risk

The relationships between anthropometric indices including WC, WHtR, BMI, VAI, ABSI, and BRI and incident T2D risk in men and women are shown in Table 2. For ABSI, no significant association was observed in either men (*P* for trend = 0.098) or women (*P* for trend = 0.313).WC, WHtR, BMI, VAI, and BRI were significantly associated with increased risk of T2D in both men and women after adjustment for potential confounders including age, smoking, drinking, physical activity and education. Further adjustment for hypertension, hyperlipidemia (except VAI), FBG (except VAI) and family history of T2D reduced the associations but still remained significant. In men, the hazard risks (95% CI; top vs lowest) were 1.43 (1.14–1.78) for WC, 1.43 (1.22–1.82) for WHtR, 2.59 (1.91–3.53) for BMI, 2.00 (1.49–2.67) for VAI, 0.79(0.61, 1.03) for ABSI, and 1.60(1.20–2.13) for BRI. For women, the corresponding HRs (95% CI) were 1.46 (1.11–1.92), 1.51 (1.10–2.09), 2.01(1.41–2.87), 1.85 (1.31–2.61), 0.83(0.61, 1.41), and 1.73 (1.21–2.46) for WC, WHtR, BMI, VAI, ABSI and BRI respectively.

**Table 2:** Anthropometric indices and incident T2D risk in men and women

Variables	Cases/Total(n)	Incidence/1000 person-years	Model 1	Model 2	Model 3
Men (*n* = 5998)					
WC (cm)					
< 85	142/3356	9.79	1.00(Reference)	1.00(Reference)	1.00(Reference)
≥ 85	223/2642	19.57	1.75(1.42,2.16)	1.73(1.40,2.14)	1.43(1.14,1.78)
*P* value			< 0.001	< 0.001	< 0.001
WHtR					
< 0.5	106/2763	8.88	1.00(Reference)	1.00(Reference)	1.00(Reference)
≥ 0.5	259/3235	18.55	1.91(1.52,2.39)	1.88(1.50,2.36)	1.43(1.22,1.82)
*P* value			< 0.001	< 0.001	< 0.001
BMI (kg/m^2^)					
< 24	98/2868	7.94	1.00(Reference)	1.00(Reference)	1.00(Reference)
24–27.9	179/2467	16.71	2.09(1.63,2.67)	2.10(1.64,2.70)	1.56(1.21,2.02)
≥ 28	88/633	30.82	4.18(3.14,5.58)	4.21(3.14,5.64)	2.59(1.91,3.53)
*P* for trend			< 0.001	< 0.001	< 0.001
VAI					
Tertile 1	78/2000	8.99	1.00(Reference)	1.00(Reference)	1.00(Reference)
Tertile 2	99/1999	11.41	1.24(0.92,1.67)	1.24(0.92,1.67)	1.17(0.86,1.58)
Tertile 3	188/1999	22.00	2.39(1.84,3.11)	2.40(1.84,3.13)	2.00(1.49,2.67)
*P* for trend			< 0.001	< 0.001	< 0.001
ABSI					
Tertile 1	119/1999	13.73	1.00(Reference)	1.00(Reference)	1.00(Reference)
Tertile 2	128/1999	14.76	0.88(0.68,1.23)	0.86(0.67,1.10)	0.80(0.62,1.03)
Tertile 3	118/2000	13.79	0.78(0.60,1.01)	0.76(0.59,0.99)	0.79(0.61,1.03)
*P* for trend			0.056	0.039	0.098
BRI					
Tertile 1	93/1999	10.79	1.00(Reference)	1.00(Reference)	1.00(Reference)
Tertile 2	109/2000	12.57	1.43(1.06,1.92)	1.42(1.06,1.91)	1.14(0.84,1.54)
Tertile 3	183/1999	21.26	2.32(1.77,3.05)	2.27(1.73,2.99)	1.60(1.20,2.13)
*P* for trend			< 0.001	< 0.001	< 0.001
Women (*n* = 3964)					
WC (cm)					
< 80	78/1899	9.50	1.00(Reference)	1.00(Reference)	1.00(Reference)
≥ 80	171/2065	18.96	1.67(1.28,2.18)	1.67(1.27,2.20)	1.46(1.11,1.92)
*P* value			< 0.001	< 0.001	< 0.001
WHtR					
< 0.5	49/1395	8.11	1.00(Reference)	1.00(Reference)	1.00(Reference)
≥ 0.5	200/2569	17.87	1.87(1.37,2.56)	1.85(1.34,2.54)	1.51(1.10,2.09)
*P* value			< 0.001	< 0.001	< 0.001
BMI (kg/m^2^)					
< 24	66/1864	8.15	1.00(Reference)	1.00(Reference)	1.00(Reference)
24–27.9	121/1486	18.70	2.31(1.71,3.11)	2.29(1.69,3.10)	1.74(1.27,2.36)
≥ 28	62/614	23.30	2.78(1.97,3.94)	2.81(1.98,4.00)	2.01(1.41,2.87)
*P* for trend			< 0.001	< 0.001	< 0.001
VAI					
Tertile 1	50/1322	8.77	1.00(Reference)	1.00(Reference)	1.00(Reference)
Tertile 2	97/1320	16.79	1.90(1.35,2.67)	1.86(1.32,2.63)	1.80(1.28,2.55)
Tertile 3	102/1322	17.72	2.04(1.45,2.87)	2.01(1.43,2.83)	1.85(1.31,2.61)
*P* for trend			< 0.001	< 0.001	0.003
ABSI					
Tertile 1	85/1321	14.74	1.00(Reference)	1.00(Reference)	1.00(Reference)
Tertile 2	79/1321	13.77	0.79(0.58,1.07)	0.77(0.56,1.05)	0.73(0.53,1.00)
Tertile 3	85/1322	14.82	0.76(0.56,1.03)	0.74(0.54,1.01)	0.83(0.61,1.41)
*P* for trend			< 0.001	0.073	0.313
BRI					
Tertile 1	46/1323	8.02	1.00(Reference)	1.00(Reference)	1.00(Reference)
Tertile 2	82/1327	14.05	1.50(1.04,2.16)	1.42(0.98,2.05)	1.23(0.85,1.78)
Tertile 3	121/1314	21.38	2.18(1.54,3.08)	1.98(1.39,2.82)	1.73(1.21,2.46)
*P* for trend			< 0.001	< 0.001	0.001

Model 1 unadjusted model. Model 2 additionally adjusted for age, smoking, drinking, physical activity and education level. Model 3 additionally adjusted for hypertension, hyperlipidemia (except VAI), FBG (except VAI) and family history of T2D

### ROC analysis

**Fig. 1:**
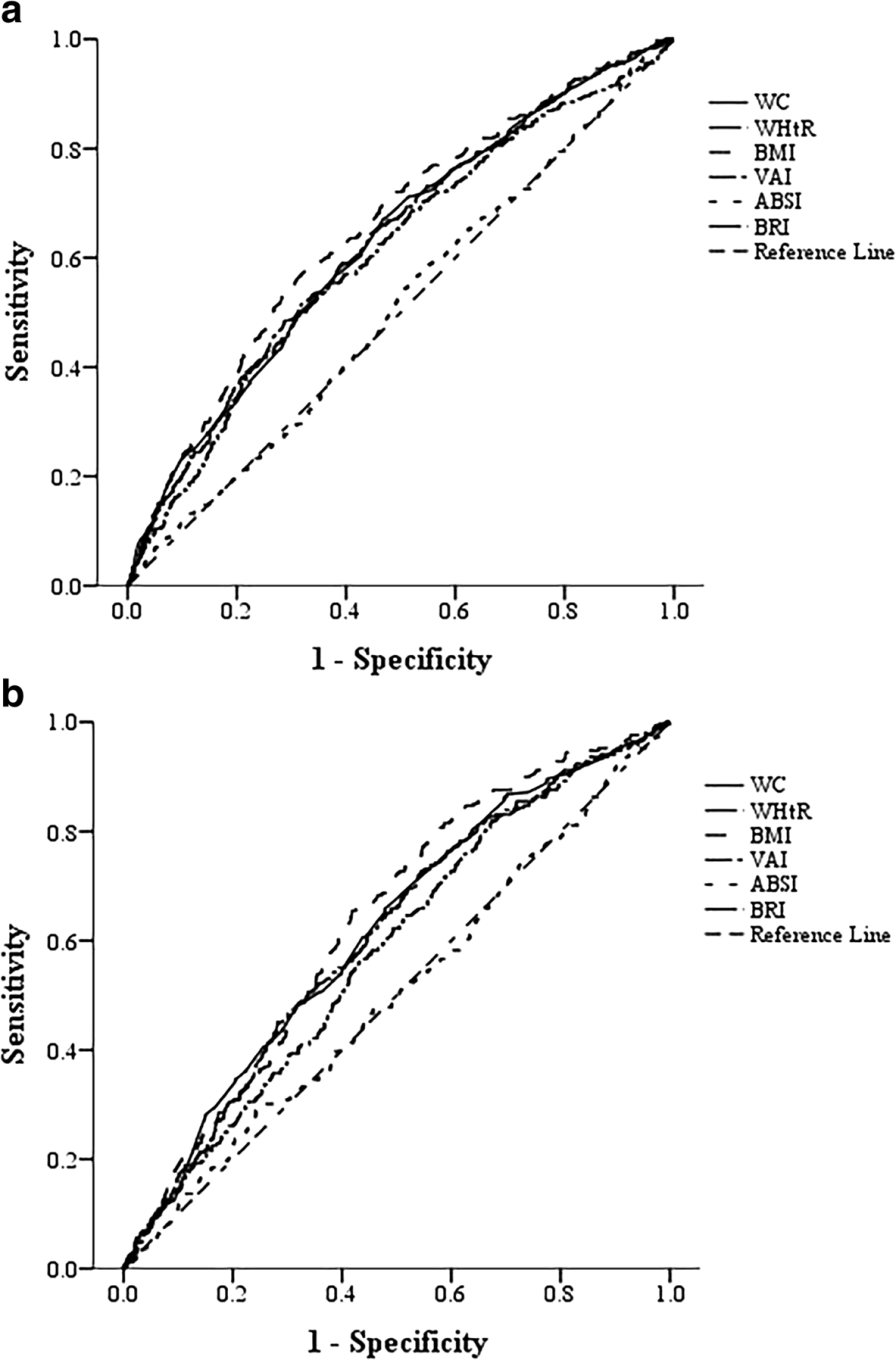
ROC curves for WC, WHtR, BMI, VAI, ABSI and BRI in men (**a**) and women (**b**) The ROC curves were constructed for T2D as a response to each anthropometric index. In men (**a**): WC: AUC = 0.629 (95% CI: 0.600–0.659). WHtR: AUC = 0.629 (95% CI: 0.600–0.658). BMI: AUC = 0.655 (95% CI: 0.626–0.684). VAI: AUC = 0.609 (95% CI: 0.578–0.639). ABSI: AUC = 0.507 (95% CI: 0.477–0.538). BRI: AUC = 0.629 (95% CI: 0.600–0.658). In women (**b**): WC: AUC = 0.616 (95% CI: 0.581–0.651). WHtR: AUC = 0.609 (95% CI: 0.574–0.644). BMI: AUC = 0.635 (95% CI: 0.602–0.667). VAI: AUC = 0.582 (95% CI: 0.548–0.617). ABSI: AUC = 0.503 (95% CI: 0.465–0.540). BRI: AUC = 0.609 (95% CI: 0.574–0.644)

Figure 1 and Table 3 presents the results of ROC analysis and AUC (95% CIs) for BMI, WC, WHtR, VAI, ABSI, and BRI. Comparison of AUC in different anthropometric indices is shown in (Additional file 1: Table S2). In men, ROC analysis revealed that BMI was the strongest predictor in diabetes (AUC = 0.655) comparable with the other anthropometric indices (*P* < 0.05) including WC (AUC =0.629), WHtR (AUC =0.629), VAI (AUC = 0.609) and BRI (AUC = 0.629). Similar as men, BMI was the strongest predictor (AUC = 0.635) in women. Except for WC (AUC = 0.616, *P* = 0.165 vs. BMI), the AUC of BMI was larger than other indices such as WHtR (AUC = 0.609, *P* = 0.051 vs. BMI), VAI (AUC = 0.582, *P* = 0.017 vs. BMI) and BRI (AUC = 0.609, *P* = 0.051vs. BMI). In contrast, ABSI was not a good predictor in either men (AUC = 0.507) or women (AUC = 0.503).

**Table 3:** AUC and corresponding 95% CI of anthropometric indices in men and women

Variables	Men (*n* = 5998)		Women (*n* = 3964)	
	AUC (95% CI)	*P* Value	AUC (95% CI)	*P* value
WC	0.629^*^(0.600,0.659)	< 0.001	0.616 (0.581,0.651)	< 0.001
WHtR	0.629^*^(0.600,0.658)	< 0.001	0.609^**^(0.574,0.644)	< 0.001
BMI	0.655 (0.626,0.684)	< 0.001	0.635 (0.602,0.667)	< 0.001
VAI	0.609^*^(0.578,0.639)	< 0.001	0.582^*^ (0.548,0.617)	< 0.001
ABSI	0.507^*^(0.477,0.538)	0.631	0.503^*^(0.465,0.540)	0.882
BRI	0.629^*^(0.600,0.658)	< 0.001	0.609^**^(0.574,0.644)	< 0.001

*AUC* area under curve, *CI* confidence interval

^*^Compared with the AUC of BMI, *p <* 0.05

^**^ Compared with the AUC of BMI, *p* = 0.051

### Optimal cutoff points of anthropometric indices in T2D risk prediction

Table 4 summarized the optimal cutoff points of the five significant anthropometric indices in prediction of T2D risk (*P* < 0.05 vs. AUC = 0.50). For men, optimal WC cut-off point was 84.90 cm in terms of Youden index; for women, the optimal cut-off point was 81.10 cm. The optimal cut-off point of BMI in men was 25.78 kg/m^2^ and 24.86 kg/m^2^ in women. The cut-off points for WHtR were similar in men (0.512) and women (0.514). For VAI, it was 1.34 in men and 1.23 in women. The optimal cut-off point of BRI in men was 3.58 and 3.62 in women.

**Table 4:** Cutoff points for anthropometric indices in predicting type 2 diabetes

Variables	Men				Women			
	Cutoff	Sensitivity (%)	Specifity (%)	Youden index	Cutoff	Sensitivity (%)	Specifity (%)	Youden index
WC (cm)	84.90	67.1	53.2	0.203	81.10	65.9	52.1	0.180
BMI (kg/m^2^)	25.78	54.2	71.3	0.255	24.86	65.5	58.2	0.237
WHtR	0.512	64.4	55.5	0.199	0.514	72.7	45.6	0.183
VAI	1.335	51.2	68.6	0.198	1.225	81.9	32.1	0.146
BRI	3.584	64.7	55.2	0.199	3.618	72.7	45.6	0.183

## Discussion

To the best of our knowledge, this is the largest prospective study to compare the novel anthropometric indices with the traditional indices in the risk prediction of T2D in elderly population. BMI, WC, WHtR, VAI, BRI were positively associated with incident T2D risk in the elderly, independent of the potential confounders. For ABSI, no significant association was observed in either men or women. BMI appeared to be the strongest predictor of incident T2D risk in both elderly men and women. In comparison with traditional anthropometric indices, novel anthropometric indices did not improve prediction of T2D in elderly population.

In the present study, BMI was the strongest predictor in both elderly men and women, while WC and WHtR showed similar prediction ability in men. These results were consistent with other studies. In Pima Indians, BMI was the best predictors of diabetes [18]. In the Health Professionals Follow-Up Study, BMI and WC showed similar associations with T2D in men aged 40–75 years; in contrast, WHR was the weakest predictor [19]. In the Atherosclerosis Risk in Communities (ARIC) Study, BMI, WC, and WHR showed similar associations in adults aged 40–64 years [20].

A large amount of studies examined the associations of novel anthropometric indices such as VAI, ABSI and BRI with diabetes risk and their performances on diabetes risk prediction. In cross-sectional [21, 22] and cohort studies [23, 24], VAI was associated with increased risk for T2D. The result was validated in the Tehran Lipid and Glucose cohort study [25]. Besides, researchers observed that VAI was a useful surrogate marker to identify risk of diabetes [23, 24], but whether the ability of the VAI to identify diabetes risk was superior to easily measurable anthropometric markers, such as BMI, WC, WHtR was still a matter of debate [22, 23, 25].

For ABSI, no significant association was observed in either men or women. ABSI was not a good discriminator of T2D in the present study. Some studies observed that ABSI was positively correlated with mortality from cardiovascular diseases and cancer [26]. Studies investigating the ABSI to predict T2D are scarce, especially in elderly population. Furthermore, until now studies have not shown that ABSI is superior to BMI or waist circumference in predicting T2D.

BRI was significantly associated with increased risk of diabetes and potential for use as an alternative index in assessment of T2D, which was consistent with previous study [11]. Till now no comprehensive consensus has been reached which one is the best anthropometric index to evaluate the risk and the predictive ability of diabetes in elderly Chinese. Whereas, compared with other anthropometric indices, BMI was taken as a useful indicator for measuring obesity in epidemiological surveys for its simplicity [3]. Our study confirmed that increased BMI was associated with type 2 diabetes in elderly Chinese population, which could be useful in initiating early interventional measures including balanced diet and regular physical exercise to prevent overweight, obesity and type 2 diabetes in the elderly population [27].

Our study also proposed optimal cut-off points for these anthropometric indices. An obvious difference was observed in WC, VAI and BRI between men and women, suggesting that gender-specific reference values should be recommended in practice. The appropriate cutoff points to best identify T2D were not consistent among different age ranges and different population. Aging not only promotes increased body fat, but also changes its distribution. Furthermore, although numerous studies proposed alternative BMI criteria specific to Asian populations [28], no consistent findings were obtained. Our ROC analysis suggested that the ideal BMI cutoffs were 25.78 kg/m^2^ and 24.86 kg/m^2^ for identifying diabetes risk in Chinese elderly men and women, respectively. Besides, in the present study WC threshold was about 85 cm for men and 81 cm for women, similar as the findings from a meta-analysis in China with WC cut-off points of 85 cm in men and 80 cm in women. While in Japan the optimal WC cutoff for abdominal obesity in men and women was 85 and 90 cm [29]. Thus, WC cutoff points cannot be used universally across gender, race or different age ranges [30]. As for VAI or BRI, there was no study about its cutoff points previously. Therefore, the definition of obesity among the elderly is still a matter of debate and our findings still need further verification.

To our knowledge, it is the first prospective study focused on elderly Chinese to comparing the novel indices with the traditional indices for diabetes risk prediction. Moreover, a notable strength was its prospective design, sex-specific analysis and relative large sample size. The study also has some limitations. Firstly, the duration of follow-up (mean 4.6 years) was relatively short and limited T2D cases were identified. Secondly, although a variety of relevant confounding factors were controlled, residual confounders could not be eliminated.

## Conclusions

In conclusion, significant associations were observed between BMI, WC, WHtR, VAI and BRI and incident risk of T2D in both elderly men and women. BMI was the strongest and best predictor of incident diabetes in elderly population. In comparison with traditional anthropometric indices, novel anthropometric indices did not improve prediction of T2D in elderly Chinese. Future studies should examine this issue in much larger samples and different population. The underlying mechanism also need to be further elucidated. Given that anthropometric indices are potentially modifiable factors, our findings may provide important public health implications for the prevention and management of T2D, especially in elderly population.

## Additional file

Additional file 1: **Table S1.** Age-adjusted correlation coefficient among anthropometric indices in men and women. **Table S2.** Comparison of AUC among different anthropometric indices in men and women. **Figure S1.** Flowchart of the participants included in the present analysis. (PDF 91 kb)

## Abbreviations

ABSI: A body shape index
ARIC: Atherosclerosis Risk in Communities
AUC: Area under curve
BMI: Body mass index
BRI: Body round index
CI: Confidence interval
DBP: Diastolic blood pressure
EDTA: Ethylene diamine tetraacetic acid
FBG: Fasting glucose concentration
HDL-c: High-density lipoprotein cholesterol
HR: Hazard ratio
LDL-c: Low-density lipoprotein
OGTT: Oral glucose tolerance test
SBP: Systolic blood pressure
T2D: Type 2 diabetes
TC: Total cholesterol
TG: Triglyceride
VAI: Visceral adiposity index
WC: Waist circumstance
WHO: World Health Organization
WHtR: Waist-to-height ratio
