# Supplementary material for: Using different anthropometric indices to assess prediction ability of type 2 diabetes in elderly population: a 5 year prospective study
Source: BMC Geriatr. 2018 Sep 17;18:218. doi: 10.1186/s12877-018-0912-2 (PMC6142386; doi:10.1186/s12877-018-0912-2)
Supplement: Supplementary file 1 — Table S1. Age-adjusted correlation coefficient among anthropometric indices in men and women. Table S2. Comparison of AUC among different anthropometric indices in men and women. Figure S1. Flowchart of the participants included in the present analysis. (PDF 91 kb) [file 12877_2018_912_MOESM1_ESM.pdf]

Table S1.Age-adjusted correlation coefficient among anthropometric indices in men and women

Variables	WC	WHtR	BMI	VAI	ABSI	BRI
**Men (n=5998)**						
WC	1.000	0.946*	0.772*	0.341*	0.573*	0.941*
WHtR		1.000	0.789*	0.328*	0.550*	0.997*
BMI			1.000	0.297*	-0.047*	0.786*
VAI				1.000	0.152*	0.325*
ABSI					1.000	0.546*
BRI						1.000
**Women (n=3964)**						
WC	1.000	0.950*	0.717*	0.259*	0.548*	0.945*
WHtR		1.000	0.729*	0.240*	0.534*	0.996*
BMI			1.000	0.160*	-0.161*	0.721*
VAI				1.000	0.165*	0.235*
ABSI					1.000	0.536*
BRI						1.000

*P<0.001.

Table S2. Comparison of AUC among different anthropometric indices in men and women

Variables	WHtR	BMI	VAI	ABSI	BRI
**Men (n=5998)**					
WC	0.958	0.015	0.208	<0.001	0.958
WHtR		0.009	0.220	<0.001	0.387
BMI			0.010	<0.001	0.009
VAI				<0.001	0.220
ABSI					<0.001
**Women (n=3964)**					
WC	0.284	0.165	0.109	<0.001	0.284
WHtR		0.051	0.203	<0.001	0.288
BMI			0.017	<0.001	0.051
VAI				<0.001	0.203
ABSI					<0.001

DeLong *et al*'s non-parametric approach was used to compare the AUCs of different anthropometric indices. The number presented in the table is *P* value.

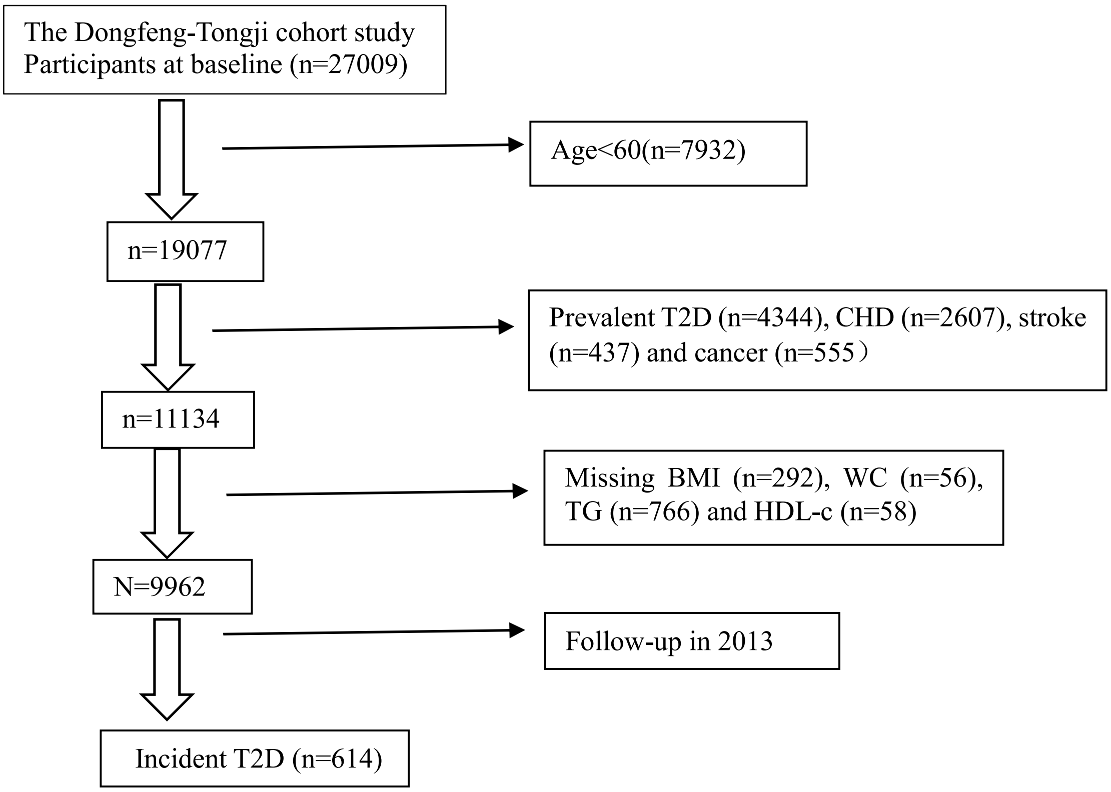

Figure S1. Flowchart of the participants included in the present analysis
